# Supplementary material for: The Small RNA Universe of Capitella teleta
Source: Front Mol Biosci. 2022 Feb 25;9:802814. doi: 10.3389/fmolb.2022.802814 (PMC8915122; doi:10.3389/fmolb.2022.802814)
Supplement: Supplementary file 1 [file DataSheet1.ZIP › Supplement/known/CAPTEscaffold_105_9392.pdf]

Provisional ID	:	CAPTEscaffold_105_9392
Score total	:	6946.5
Score for star read(s)	:	3.9
Score for read counts	:	6936.8
Score for mfe	:	1.2
Score for randfold	:	1.6
Score for cons. seed	:	3
Total read count	:	13618
Mature read count	:	12772
Loop read count	:	0
Star read count	:	846

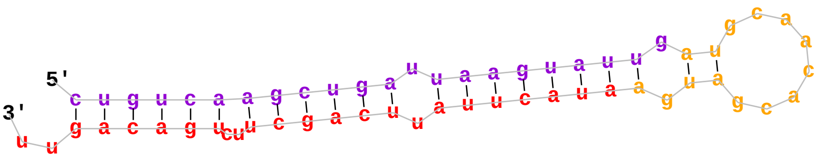

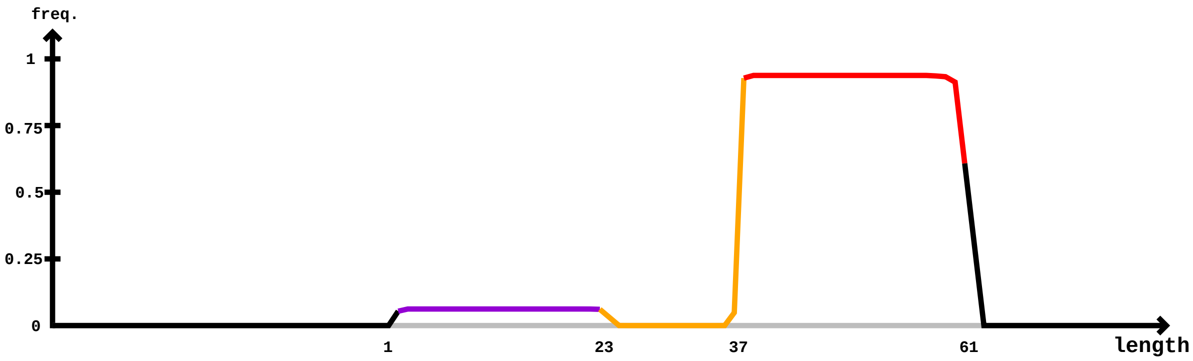

Star **Mature**

```
5'- ugaauuggccauuggugaacuguguguaaagugccugucaagcugauuaaguauugaugcaacacgaugaauacuuauucagcuucugacaguugguuuccacaacugaacaac  -3'  obs
    ugaauuggccauuggugaacuguguguaaagugccugucaagcugauuaaguauugaugcaacacgaugaauacuuauucagcuucugacaguugguuuccacaacugaacaac       exp
    ugaauuggccauuggugaacuguguguaaagugccugucaagcugauuaaguauugaugcaacacgaugaauacuuauucagcuucugacaguugguuuccacaacugaacaac       known
    ....((.(((...))).))...((((.(((.((.((((((((((((.((((((((.((........)).)))))))).))))))..)))))).)).))).))))..........   reads   mm   sample

    ..................................cugucaagcugauuaaguau............................................................   1   0   seq
    ..................................cugucaagcugauuaaguauu...........................................................   15  0   seq
    ..................................cugucaGgcugauuaaguauug..........................................................   1   1   seq
    ..................................cAgucaagcugauuaaguauug..........................................................   1   1   seq
    ..................................cugucaagcGgauuaaguauug..........................................................   1   1   seq
    ..................................cugucaagcuAauuaaguauug..........................................................   1   1   seq
    ..................................cugucaagcugauuaagCauug..........................................................   1   1   seq
    ..................................cugucaagcAgauuaaguauug..........................................................   1   1   seq
    ..................................cugucaagcugauuaagAauug..........................................................   2   1   seq
    ..................................cugucaagcugauuaaguauug..........................................................   705 0   seq
    ..................................cugucaagcugauuUaguauug..........................................................   1   1   seq
    ..................................cuAucaagcugauuaaguauug..........................................................   1   1   seq
    ..................................cugucaagcugaAuaaguauug..........................................................   1   1   seq
    ..................................cugucaagAugauuaaguauug..........................................................   1   1   seq
    ..................................cugucaagcugauuaaguauugaA........................................................   1   1   seq
    ...................................ugucaagcugauuaaguau............................................................   1   0   seq
    ...................................ugucaagcugaCuaaguauug..........................................................   1   1   seq
    ...................................uUucaagcugauuaaguauug..........................................................   1   1   seq
    ...................................ugucaagcugauuaaguauug..........................................................   73  0   seq
    ...................................ugucaagcugauuaaguauuga.........................................................   35  0   seq
    ...................................ugucaGgcugauuaaguauuga.........................................................   1   1   seq
    ....................................................................Caauacuuauucagcuucugacagu.....................   1   1   seq
    .....................................................................aauacuuauucagcuucugac........................   26  0   seq
    .....................................................................aauacuuauAcagcuucugaca.......................   1   1   seq
    .....................................................................aauacuuauucagcuucugaca.......................   4   0   seq
    .....................................................................aauacuuauucagcuucugacGg......................   1   1   seq
    .....................................................................aauacuuauucagcuucugacag......................   16  0   seq
    .....................................................................aauacuuauucagcuucugacagG.....................   1   1   seq
    .....................................................................aauacuuauucagcuucugacaUu.....................   1   1   seq
    .....................................................................aauacuuauucagcAucugacagu.....................   1   1   seq
    .....................................................................aauacuuaAucagcuucugacagu.....................   1   1   seq
```

Star Mature

```
ugaauuggccauuggugaacuguguguaaagugccugucaagcugauuaaguauugaugcaacacgaugaauacuuauucagcuucugacaguugguuuccacaacugaacaac
.....................................................................aauacuuauucagcuucugacagu.....................  579   0   seq
.....................................................................aauacuuauucagAuucugacagu.....................  1     1   seq
.....................................................................Nauacuuauucagcuucugacagu.....................  1     1   seq
.....................................................................aauacuuauucagcuucugacagA.....................  1     1   seq
.....................................................................aauacuuauAcagcuucugacagu.....................  1     1   seq
.....................................................................aauacuuauuUagcuucugacagu.....................  1     1   seq
.....................................................................aaAacuuauucagcuucugacagu.....................  2     1   seq
.....................................................................aauacuuauucagcuucugacagC.....................  1     1   seq
.....................................................................aauacuuauucGgcuucugacagu.....................  1     1   seq
.....................................................................aauacuuauucagcuucugacaguu....................  14    0   seq
.....................................................................Cauacuuauucagcuucugacaguu....................  4     1   seq
......................................................................auacuuGuucagcuucugaca.......................  3     1   seq
......................................................................auacCuauucagcuucugaca.......................  1     1   seq
......................................................................auacuuauucagcuucugaca.......................  30    0   seq
......................................................................auacuuauucagcuucCgacag......................  1     1   seq
......................................................................auacuuGuucagcuucugacag......................  5     1   seq
......................................................................auacuuauucagAuucugacag......................  1     1   seq
......................................................................auacuuauucagcuucugGcag......................  1     1   seq
......................................................................auacuuauucagcuucugacag......................  247   0   seq
......................................................................auacuuauucaCcuucugacag......................  1     1   seq
......................................................................auacuuauucagcuucugacaAu.....................  1     1   seq
......................................................................auacuuauucagcuucugaAagu.....................  3     1   seq
......................................................................Uuacuuauucagcuucugacagu.....................  1     1   seq
......................................................................auacuuauucagcuucugGcagu.....................  1     1   seq
......................................................................auacuuauuAagcuucugacagu.....................  10    1   seq
......................................................................aNacuuauucagcuucugacagu.....................  1     1   seq
......................................................................auacuuauucCgcuucugacagu.....................  1     1   seq
......................................................................auacuuauucagcuucuAacagu.....................  2     1   seq
......................................................................aCacuuauucagcuucugacagu.....................  1     1   seq
......................................................................aAacuuauucagcuucugacagu.....................  4     1   seq
......................................................................auacuuauucagcuucugacagA.....................  2     1   seq
......................................................................auGcuuauucagcuucugacagu.....................  1     1   seq
......................................................................auacuuauucagcuucugacagu.....................  3464  0   seq
......................................................................auacuuauucagcuucuUacagu.....................  1     1   seq
......................................................................auacuuauucGgcuucugacagu.....................  4     1   seq
......................................................................auacuuauuGagcuucugacagu.....................  2     1   seq
......................................................................auacAuauucagcuucugacagu.....................  5     1   seq
......................................................................auacuuGuucagcuucugacagu.....................  28    1   seq
......................................................................auacuuauucagUuucugacagu.....................  1     1   seq
......................................................................Guacuuauucagcuucugacagu.....................  2     1   seq
......................................................................auacuuauAcagcuucugacagu.....................  1     1   seq
......................................................................auacuuauucagcuucugaUagu.....................  1     1   seq
......................................................................auacuuauucagcuAcugacagu.....................  1     1   seq
......................................................................auacuuauucagcCucugacagu.....................  1     1   seq
......................................................................auacuuaAucagcuucugacagu.....................  2     1   seq
......................................................................auacuuauuUagcuucugacagu.....................  9     1   seq
......................................................................auacuuauucagcuuUugacagu.....................  1     1   seq
......................................................................auacuuauucagcuucugacagG.....................  2     1   seq
......................................................................auacuuauucagcuucCgacagu.....................  1     1   seq
......................................................................auacuuauucagcuucugUcaguu....................  3     1   seq
......................................................................auacuuauucagcuucugacGguu....................  3     1   seq
......................................................................auacuuauucagcuucugaUaguu....................  1     1   seq
......................................................................auacuuauucagcuucugacCguu....................  1     1   seq
......................................................................aAacuuauucagcuucugacaguu....................  3     1   seq
......................................................................auacuuauucagcuucugacagAu....................  3     1   seq
......................................................................auacuuauucagcuucuAacaguu....................  3     1   seq
......................................................................auacuuUuucagcuucugacaguu....................  1     1   seq
......................................................................auacuuauucagcAucugacaguu....................  2     1   seq
......................................................................auacuuauucagcuucugacaAuu....................  1     1   seq
......................................................................auacuuauucaUcuucugacaguu....................  2     1   seq
......................................................................auacuuGuucagcuucugacaguu....................  14    1   seq
......................................................................auaAuuauucagcuucugacaguu....................  1     1   seq
......................................................................auacAuauucagcuucugacaguu....................  9     1   seq
......................................................................auacuuauCcagcuucugacaguu....................  2     1   seq
......................................................................auGcuuauucagcuucugacaguu....................  1     1   seq
......................................................................auacuAauucagcuucugacaguu....................  4     1   seq
......................................................................auacuuauucagcuucugGcaguu....................  1     1   seq
......................................................................auacuuauucagcuucGgacaguu....................  1     1   seq
......................................................................auacuuauucaCcuucugacaguu....................  1     1   seq
......................................................................auacuuauucagcuucugaGaguu....................  2     1   seq
```

```
                                  Star                                Mature
ugaauuggccauuggugaacuguguguaaagugccugucaagcugauuaaguauugaugcaacacgaugaauacuuauucagcuucugacaguugguuuccacaacugaacaac
......................................................................auacuuauucagAuucugacaguu....................   5      1     seq
......................................................................auacuuauucagcuGcugacaguu....................   2      1     seq
......................................................................auacuuauucagcuucugacaguG....................   6      1     seq
......................................................................auacuuauucagcuucugaAaguu....................   1      1     seq
......................................................................auacuuauucagcuucugacaguA....................   3      1     seq
......................................................................auacuuauuAagcuucugacaguu....................   17     1     seq
......................................................................auacuuauuUagcuucugacaguu....................   8      1     seq
......................................................................auacuuauucagcuucAgacaguu....................   6      1     seq
......................................................................auacuuauucagcCucugacaguu....................   3      1     seq
......................................................................auacuuauucagcuCcugacaguu....................   1      1     seq
......................................................................auacuuauucagcGucugacaguu....................   1      1     seq
......................................................................auacuuauucagcuucugacaguu....................   7901   0     seq
......................................................................Nuacuuauucagcuucugacaguu....................   3      1     seq
......................................................................auacuuauucGgcuucugacaguu....................   3      1     seq
......................................................................auacuuauAcagcuucugacaguu....................   3      1     seq
......................................................................Guacuuauucagcuucugacaguu....................   3      1     seq
......................................................................auacuuauucUgcuucugacaguu....................   1      1     seq
......................................................................auacuuaAucagcuucugacaguu....................   8      1     seq
......................................................................Uuacuuauucagcuucugacaguu....................   2      1     seq
......................................................................auacuCauucagcuucugacaguu....................   2      1     seq
......................................................................auacuuauucagcuucugacaguuU...................   73     1     seq
......................................................................auacuuauucagcuucugacaguuA...................   22     1     seq
.......................................................................uacuuauucagcuucugacag......................   3      0     seq
.......................................................................uacuuauucagcuucugacagu.....................   4      0     seq
.......................................................................uacuuauucagcuucugacaguu....................   95     0     seq
.......................................................................uacuuauuAagcuucugacaguu....................   1      1     seq
.......................................................................uacuuauucagcuucugacaguuU...................   5      1     seq
.......................................................................uacuuauucagcuucugacaguuA...................   28     1     seq
```
